# The Prenatal Microbiome: A New Player for Human Health

**DOI:** 10.3390/ht7040038

**Published:** 2018-12-11

**Authors:** Valeria D’Argenio

**Affiliations:** 1 CEINGE-BiotecnologieAvanzate, via G. Salvatore via G. Salvatore 486, 80145 Naples, Italy; dargenio@ceinge.unina.it; Tel.: +39-081-373-7909; 2 Department of Molecular Medicine and Medical Biotechnologies, University of Naples Federico II, via Pansini 5, 80131 Naples, Italy; 3 Task Force on Microbiome Studies, University of Naples Federico II, via Pansini 5, 80131 Naples, Italy

**Keywords:** prenatal microbiome, healthy status, metagenomics, fetal development

## Abstract

The last few years have featured an increasing interest in the study of the human microbiome and its correlations with health status. Indeed, technological advances have allowed the study of microbial communities to reach a previously unthinkable sensitivity, showing the presence of microbes also in environments usually considered as sterile. In this scenario, microbial communities have been described in the amniotic fluid, the umbilical blood cord, and the placenta, denying a dogma of reproductive medicine that considers the uterus like a sterile womb. This prenatal microbiome may play a role not only in fetal development but also in the predisposition to diseases that may develop later in life, and also in adulthood. Thus, the aim of this review is to report the current knowledge regarding the prenatal microbiome composition, its association with pathological processes, and the future perspectives regarding its manipulation for healthy status promotion and maintenance.

## 1. Introduction

The human microbiome has emerged as an important factor required for human health status acquisition and maintenance [1,2]. Indeed, several reports have demonstrated that microbial alterations (or dysbiosis) can be found in an increasing number of human diseases and that their entities may be associated with the severity of the phenotype or with the sensitivity to specific therapies [1,2,3,4,5,6,7,8]. Thus, even if it has not been well established yet whether the microbiome alterations are the cause or the consequence of diseases, their study is intriguing since it opens the way to the identification of novel disease-specific biomarkers, or to the development of targeted therapies aiming to manipulate the microbiome in the attempt to restore a healthy status [9,10,11,12,13,14,15,16,17]. Next generation sequencing-based approaches have assessed their reliability in metagenomic studies escalating the possibility to describe microbial communities in detailand obtain both qualitative and quantitative information [1,2,18,19]. As a consequence, it is now widely accepted that microbes live on the surface and inside our body, colonizing also body niches that were traditionally considered as “sterile.” This applies also to the fetal environment [20,21,22]. Indeed, technological advances in metagenomics have denied a central dogma of reproductive medicine stating that the fetus stays in a sterile niche (i.e., the uterus) and is colonized by bacteria only at birth [23]. The prenatal microbiome seems not only to have a role in fetal development but may also influence the subsequent adult health status [24,25,26]. Thus, understanding fetal microbiome origins and the factors able to influence its composition is important to clarify diseases pathogenesis and identify also maternal factors that, acting at a prenatal age, may predispose the development of diseases including later in adulthood [24,25,26]. The aim of this review is to describe the knowledge on prenatal microbiome, its association to specific diseases, and its possible manipulation to promote a healthy status.

## 2. The Prenatal Microbiome

For about a century, it has been assumed that the fetus is sterile and that microbes colonize the newborn immediately during birth, withthe delivery mode being an important modifying factor [23,27,28]. The placenta, in particular, has been considered for years like a barrier able to prevent fetal contamination by maternal toxins and microbes, thus preserving fetal sterility and ensuring proper fetal development [26,29,30]. In contrast with these beliefs, recent studies have shown that microbes colonize the amniotic fluid, the umbilical blood cord, and the placenta, suggesting that a maternal microbial colonization of the fetus mayalready begin in utero [20,21,22,23,24,25,26,30]. In particular, the presence of an established microbiota in healthy and term infants, in absence of any infection or inflammation, supports the hypothesis that not only microbes colonize the fetus before birth, but that they may also play a role into the physiological development of the fetus.

In support of this hypothesis, microbial communities have been found also in the meconium of newborns [31,32,33,34,35,36,37,38,39,40,41,42]. Jiménez et al. first demonstrated the non-sterility of infant meconium [31]. These authors were also able to showthe presence of a maternal–fetalin utero transmission of gut microbiota; they used a mouse model to verify that a specific *Enterococcus faecium* strain, maternally administer viaoral instillation, was present in the gut of the pups delivered 1 day prematurely bycesarean section [31]. Subsequently, several reports using different molecular strategies have described microbial meconium communities, also highlighting interesting differences between term and preterm infants [32,33,34]. In particular, Mshvildadze et al. characterized the meconium microbiome of 23 neonates, revealing an association between a reduced microbial diversityand prematurity [32]. In a prospective longitudinal study, Madanet al. found thatthe predominant bacterial genera in the meconium specimens obtained from six preterm neonates were *Lactobacillus*, *Staphylococcus*, and *Enterobacteriaceae* [33]. Ardissone et al. analyzed the meconium collected from 52 infants with gestational ages ranging from 23 to 41 weeks and found that the gestational age was correlated with low bacterial diversity and microbial colonization [34]. In addition, this study revealed similar microbial features between the meconium and the amniotic fluid, thus supporting the hypothesis of an intrauterine origin of the meconium microbiota, as a consequence of the amniotic fluid swallowing by the fetus during the last trimester of pregnancy [34]. According to these findings, Collado et al. analyzed the microbiome of multiple samples from mother–offspring pairs, including the placenta, the meconium, and the amniotic fluid, and highlighted the presence of common microbial features [35]. The meconium microbiota has features of a low biodiversity and a high inter-individual variability [36,37]. Bäckhedet al. found that in the meconium microbiota, the genera *Escherichia-Shigella* and *Enterococcus* were more abundant and *Bacteroides* and *Bifidobacterium* less abundant with respect to infants fecal microbiota [38]. Tapiainen et al. carried out a large population-based study, recruiting 218 consecutively birth newborns, to assess the role of bacterial exposures before birth on the meconium microbiome composition [39]. They found that maternal factors occurring during pregnancy affect the meconium microbiome composition supporting the hypothesis of the in utero transfer of microbes [39].

Other studies have investigated the potential impact of the delivery mode on the meconium microbiome composition [40,41,42]. By analyzing the 16S rRNA profiles of rectal swabs obtained at birth by full-term infants, Dominguez-Bello et al. found that meconium microbiota composition is influenced by delivery mode [40]. In particular, they highlighted that in cesarean-delivered infants, there was a prevalence of *Staphylococci* with respect to the vaginally-delivered babies, whose microbiome was featured by the presence of genera like *Lactobacillus* and *Prevotella*, resembling the skin microbiome, and the vaginal microbiome, respectively [40]. Other studies performed on independent cohorts of full-term infants also found that the meconium microbiota of vaginally-delivered infants is dominated by lactic acid or enteric bacteria [41,42].

**Figure 1:**
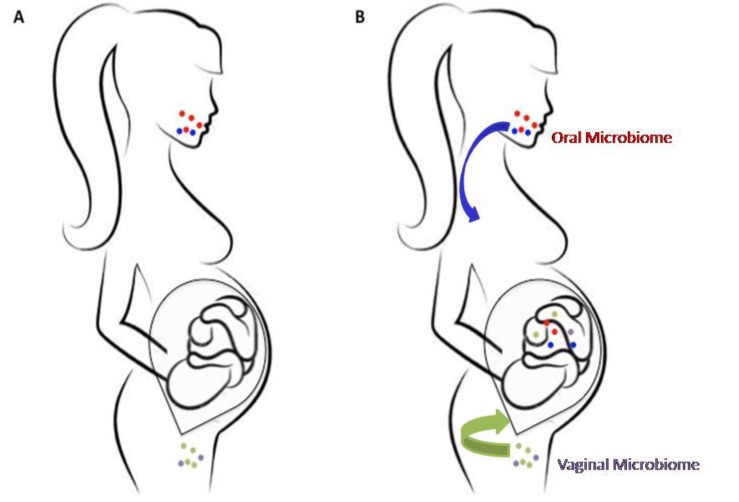
Prenatal microbiome colonization. (**A**) For years the uterus has been considered as a sterile womb in which the fetus is protected by the placenta from microbial colonization. (**B**) Recent studies have questioned this hypothesis showing that microbial colonization may already begin in utero; these microbes may reach the fetus from the maternal oral cavity/gut through the bloodstream (hematogenous route) and from the vagina (ascendant route).

Even if the origin of meconium microbiota has been not yet clearly established, these data suggest that the establishment of intestinal microbiota is initiated in the prenatal gut. Indeed, as stated above, recent evidence has overcome the traditional view of the in utero environment as being a sterile room (Figure 1A) [20,21,22].

In particular, the presence of bacteria in the placenta of preterm newborns or in case of intrauterine infections is not surprising. However, bacteria have also been found in the placenta of at term pregnancy, without any sign of infection, and also after cesarean section under sterile conditions [43]. Aagaard et al. used 16S rRNA sequencing to analyze the placenta-related microbial communities of 320 women and revealed the presence of a low-abundant, but metabolically rich microbiota, featured by the presence of bacteria belonging to the phyla Firmicutes, Tenericutes, Proteobacteria, Bacteroidetes, and Fusobacteria [44]. Bacterial communities have been found also in the amniotic fluid, even without amniotic sac ruptures [45]. Other studies have focused their attention on the umbilical cord blood: Jiménez et al. analyzed the bacterial communities of healthy, full-term newborns after elective cesarean delivery and highlighted the presence of commensal bacteria, like *Enterococcus*, *Streptococcus*, *Staphylococcus*, and *Propionibacterium* [31].

All these data support the hypothesis of a prenatal origin of human microbiome. However, the origin of this fetal microbiome is still unknown (Figure 1B). The currently accepted theory is that fetal microbial bacteria may originate both from the vaginathrough ascendant colonization [46,47,48,49,50], and from the maternal oral cavity/gut through the bloodstream (hematogenous route) [31,44,51,52,53,54,55]. Additional studies are required to better clarify these issues.

## 3. Correlation between Fetal Microbiome and Newborn’s Health

The human gut is the organ with the highest density of microbial communities [1,2,3,4,6,7,8,56]. These communities are involved in several functions required for human host healthy status [1,2,3,4,6,7,8,56]. Thus, the initial microbial colonization of human gut is a critical step able to influence the host health and diseases risk through the establishment of a number of microbes–human interactions [21,22,23,24,25,26]. Maternal and early life environmental factors can both influence the gut microbiota development and composition and, as a consequence, may increase the risk of several chronic and metabolic diseases later in life [21,22,23,24,25,26]. Indeed, maternal microbial communities, including prenatal gut, vaginal, oral, and skin microbiomes, undergo pronounced changes during pregnancy that may affect healthy status maintenance and contribute to the development of common diseases [20]. To date, a microbial dysbiosis of the infant gut has been associated to the development of asthma, allergic diseases, and obesity [21,22,23,24,25,26]. Thus, considering that it has been established that the fetus does not stay in a sterile environment and that the microbial colonization begins in utero, factors that are able to influence this first colonization may impact on newborn’s future health.

In particular, it is now clear that the fetal immune system is not inactive but, being exposed through the mother to environmental stimuli, interacts with the maternal immune system [57,58]. Indeed, several maternal factors, including hormones, cytokines, and the microbiome, can modify the intrauterine environment, thus impacting fetal immune system development [57,59,60]. The toll-like receptors (TLRs) are a class of receptors present on the surface of different cells (macrophages, mast cells, and dendritic cells) involved in the innate immunity. Since different types of TLRs are able to recognize distinct bacteria driving the development of a potential inflammatory response, it has been proposed that intrauterine bacteria may influence the fetal immune system development though the TLRs [61,62]. Another possible mechanism of bacterial influence on the fetal immune system development may be represented by the production of short chain fatty acids (SCFAs) that are able to induce T-cells activation and modulate interleukin -10 production [63,64]. Finally, mucosa-associated invariant T cells (MAIT) have also been identifiedin the fetus starting from the second trimester of pregnancy [65,66]. These cells are able to recognize microbial metabolites and, consequently, to produce inflammatory cytokines supporting the hypothesis of in utero activity [66,67].

Considering all the above, it is easy to understand that all the factors influencing maternal health, including maternal habits, during pregnancy and the partum can influence the microbial colonization of the fetus. Chu et al. evaluated the effect of maternal diet on meconium microbiome composition [68]. In this study, 81 mother-infants couples were analyzed to highlight microbial alterations related to maternal diet during pregnancy. Interestingly, meconium microbial communities were significantly different in high fat respect to balanced diet exposed infants, *Bacteroides* being significantly reduced in high-fat exposed infants [68]. Another study by Lundgren et al. aimed to verify the relationship between maternal diet during pregnancy and the stool microbiome evaluated 6 weeks after birth [69]. In total, 145 infant/mother couples were analyzed showing that several maternal dietary factors during pregnancy influenced infants’ stool microbiomes and that these alterations are also related to the delivery mode [69]. Collado et al. found that infants’microbiomes are influenced by the mother beingoverweight during pregnancy [70]. In particular, this study highlighted that in the presence of an excessive maternal weight gain during pregnancy, newborns have lower amounts of the genera *Bacteroides* and *Prevotella* with respect to those born from mothers with regular weight gain [70]. Hu et al. studied the correlation between maternal gestational and pre-gestational type 2 diabetes and the meconium microbiome composition [36]. Interestingly, they found that *Bacteroides*, *Parabacteroides*, and *Lachnospiraceae* were more abundant in the meconium of infants in the presence of maternal diabetes and that these alterations were higher in the case of pre-gestational diabetes [36]. Gosalbes et al. verified the effects of maternal atopic diseases on the meconium microbiome [41]. They found that maternal eczema is able to modify the meconium bacterial composition, being associated with a lower bacterial diversity and richness and to an increased abundance of the *Enterobacteriaceae* family, while maternal asthma or rhinitis seem to not induce microbial changes [41]. Finally, Mshvildadze et al. showed that a lower bacterial diversity was present in the meconium of infants whose mothers received intra-partum antibiotics [32]. A recent interesting study by Lammert et al. investigated the role of the prenatal microbiome on the development of behavioral alterations [71]. Using a mouse model of autism, they were able to assess that prenatal microbiota transplantation is able to modulate the maternal immune system and transfer the susceptibility to neurodevelopmental disorders [71].

These studies support the hypothesis that environmental factors during pregnancy can modify maternal microbiome and may affect the fetal environment. However, the effects of this in utero colonization on newborns future health are not yet clearly understood and further studies on large cohorts of subjects are required to investigate how environmental stimuli can impact maternal microbiome during pregnancy and the long-term effects of these perturbations on infants’ health.

## 4. Manipulation of Prenatal Microbiome for Healthy Status Maintenance

As discussed in the previous sections, recent studies have shown that microbial colonization begins already before birth and that both maternal and environmental factors may play a role in this microbial early colonization. Even if the origin of the prenatal microbiome is still poorly understood and its correlation with newborns health and disease status also needs to be further investigated, if confirmed by future studies, it may become an actionable target to promote infant health by inducing the colonization of beneficial bacteria and avoiding those considered harmful.

Indeed, unlike the human genome, the microbiome can be easily modified, and its evaluation may be useful not only to stratify individuals based on a specific disease risk, but also to monitor them overtime [72]. In addition, while inter-individual differences in the response to specific therapies are well known, few data are available regarding the role of microbiomes in these phenomena, even if it has been established that the gut microbiota is involved in the metabolic transformation of several therapeutic compounds [72]. Thus, a better understanding of these processes may drive personalized medicine by supporting the choice of the most proper treatment and minimizing the onset of side effects.

Considering all the above, there is a lot of enthusiasm in this field since microbiome gives the opportunity to have a non-invasive biomarker that can not only be used for diagnostic purposes, but is emerging as an easily actionable target for therapeutic interventions. Antibiotics, probiotics, and prebiotics administration may be easily used to induce specific microbiome modifications [73,74,75,76]. In addition, enteral nutrition and microbiome transplantation have also shown their efficacy in some specific conditions [77,78,79].

The more our knowledge on human microbiome and its role in disease pathogenesis increases, the more the possibility to treat specific conditions (or at least to ameliorate their clinical outcome) by microbiome manipulation becomes intriguing. This also applies to the prenatal microbiome: since it is influenced by maternal habits during pregnancy, it is easy to imagine possible interventions (for example on maternal diet) that are able to drive fetal microbial colonization. However, as in other fields, a lot of limitations need to be assessed before microbiome manipulation can be genuinely helpful in routine clinical assessments. Microbial inter-individual variability, technical challenges, and potential pitfalls related to samples collection, analysis, and interpretation have to be taken into account.

## 5. Conclusions

As reviewed above, in the last 10 years, the sterile womb paradigm has been changed to the in utero colonization hypothesis. Indeed, microbial communities have been identified in the meconium, the amniotic fluid, the umbilical blood cord, and the placenta in at term pregnancy and in absence of infections [21,26].

To date, several reports have attempted to study the prenatal microbiome in order to clarify its origins and functions, and consequently highlight a possible role in predisposing to diseases development also later during life and adulthood [24,25,26]. Interesting associations have emerged and these may be the bases for the possible use of the prenatal microbiome as a biomarker and also as a potentially actionable target to be manipulated for personalized treatments aimed to reduce the risk for specific diseases.

Of course, caution is required regarding the correct interpretation of these data. As recently stated by Perez-Munoz et al. concerns exist regarding the detection limits of the used techniques (PCR and next generation sequencing-based approaches do not have high enough sensitivity to study low-rich bacterial communities), and with the high risk for environmental contaminations combined with the lack of proper controls [30]. In addition, the currently used next generation sequencing-based technologies cannot discriminate between live, viable, and active *versus* dead, unviable, and inactive bacteria. All these issues need to be taken into account when considering the prenatal microbiome and its influence on future health. Maybe further studies combining traditional culture-based assays and more extensive microbial communities studies (i.e., metabolomics, metatranscriptomics, and metagenomics) may provide a more comprehensive view of the prenatal microbiome and its role. Once these points are clarified and clearly assessed, novel perspectives for personalized and predictive medicine may be available based on the knowledge of the prenatal microbiome.
